# Hemoglobin stimulates the expression of ADAMTS-5 and ADAMTS-9 by synovial cells: a possible cause of articular cartilage damage after intra-articular hemorrhage

**DOI:** 10.1186/s12891-017-1815-7

**Published:** 2017-11-14

**Authors:** Takuya Tajima, Tomohisa Sekimoto, Nami Yamaguchi, Noboru Taniguchi, Syuji Kurogi, Masugi Maruyama, Etsuo Chosa

**Affiliations:** 1 Division of Orthopaedic Surgery, Department of Medicine of Sensory and Motor Organs, Faculty of Medicine, University of Miyazaki, 5200 Kihara, Kiyotake, Miyazaki, 889-1692 Japan; 2 Department of Medical Sciences Applied Physiology, Faculty of Medicine, Graduate School of Medicine, University of Miyazaki, 5200 Kihara, Kiyotake, Miyazaki, Miyazaki 889-1692 Japan

**Keywords:** ADAMTS-5, ADAMTS-9, Articular cartilage, Hemoglobin, Synovial cells

## Abstract

**Background:**

ADAMTS (a disintegrin and metalloprotease with thrombospondin motifs) proteins play an important pathological role in matrix degeneration. Aggrecan degradation is a significant and critical event in early-stage osteoarthritis. To determine the effect of hemoglobin (Hb) on the ability of synovial tissues to produce ADAMTS family members, we examined the influence of Hb by synovial cells in an in vitro experimental system.

**Methods:**

Synovial tissues were obtained from five young patients with meniscal injury under arthroscopic surgery. Primary cultures of human knee synovial cells were treated with different doses of human Hb (0, 25, 50, 100 μg/ml). The culture media were collected 24 h after Hb-treatment. In the time-course studies, cells were treated with and without 100 μg/ml Hb, and culture media were taken at 6, 12, and 24 h. To identify the proteins responsible for aggrecanase activity, Western blot analysis using antibodies against human ADAMTS-5, −8, −9, and −10; enzyme-linked immunosorbent assay (ELISA); and gene expression for ADAMTS-5 and -9 were examined. Statistical comparisons between each group were performed using paired *t*-tests.

**Results:**

Western blot analysis revealed that Hb-treatment resulted in the expression of ADAMTS-5 and -9. Neither control group nor Hb-treated medium showed immunoreactivity against ADAMTS-8 or −10. In a dose-dependency study, the Hb-treated group showed significantly higher levels of ADAMTS-5 and -9 compared with the control (*p* < 0.05). There was no significant difference between 25, 50, and 100 μg/ml Hb-treated groups. In a time-course study, the ADAMTS-5 and -9 levels in the conditioned medium had significantly increased expression at 6, 12, and 24 h in the Hb-treated group (*p* < 0.05). Hb evoked significant expression of ADAMTS-9 mRNA at 12 and 24 h (*p* < 0.05).

**Conclusions:**

These findings indicate that Hb induces the expression of ADAMTS-5 and -9 by synovial cells at low doses, even at an acute phase, and suggests a possible role for Hb in cartilage damage after intra-articular hemorrhage. The results also suggest a new potential therapeutic target by inhibiting the activities of ADAMTS-5 and -9 to prevent cartilage damage after intra-articular hemorrhage.

## Background

Hemoarthrosis or intra-articular bleeding caused by joint trauma such as ligament rupture or intra-articular fractures leads to the various long-term problems and disorders such as joint pain, joint swelling, stiffness and decreased range of motion, joint deformity, and dysfunction [1]. Synovial tissues play important and critical roles in the situation of blood-induced joint disorders have been reported [1, 2]. In experimentally model of hemoarthrosis, proliferation and hypertrophic change of synovial tissue, neovascularization, and a perivascular acute inflammatory reaction were confirmed at the initial stage. At the end-stage of hemoarthrosis, the joint is characterized by a breakdown of articular cartilage, joint effusion, formation of fibrous tissue, and secondary osteoarthritis. On the other hand, not only repeated hemoarthrosis, but also even a single issue of intra-articular hemorrhage following trauma that frequently occurs in the cases of sports injuries, may cause irreversible joint damage. Moreover, exposure of articular cartilage to low concentrations of blood for a period as short as 2 days has also been confirmed to induce the irreversible cartilage damage [3, 4]. However, the pathomechanisms of blood-induced joint damage is still unclear in detail.

Roosendaal et al. examined the effects and mechanisms of blood-induced joint damage in vivo and in vitro study; which blood components contribute to the cartilage damage. They reported that erythrocytes play critical and important roles in blood-induced joint disorder and this effect was independent of cytokine production [5–7].

Articular cartilage is constituted of small numbers of chondrocytes which were embedded in large amount of extracellular matrix containing collagen and aggrecan. The extracellular matrix of the articular cartilage provides the essential biomechanical characteristics for joint motion. For example, the important function of resisting compressive force is provided by aggrecan, a large proteoglycan [8]. Degeneration of aggrecan is a critical event in articular cartilage destruction [9].

Aggrecanases are members of the ADAMTS family (A disintegrin and metalloprotease with thrombospondin motifs), and are the principal enzymes responsible for aggrecan cleavage in the initial events of cartilage turnover [9]. Aggrecanase-mediated aggrecan degeneration is also a key and critical event in initial-stage osteoarthritis. 19 ADAMTS genes numbered 1 to 20 were already identified in mammalian genomes; the designation ADAMTS-11 is excluded because it was already identified as ADAMTS-5, previously [10]. Many members, such as ADAMTS-1, −4, −5, 8, −9, −15, −16, and −18, have been reported to cleave aggrecan [8–12].

Previously, it has been reported that hemoglobin (Hb) stimulates urokinase type plasminogen activator (uPA), matrix metalloproteinase (MMP)-2, and MMP-9 expression by cultured fibroblasts and synovial cells, indicating that Hb contributes to the cellular functions management [4, 13]. Based on these findings, Hb may play critical and important roles to enhance the expression of proteinases from synovial cells, leading to the destruction of the extracellular matrix of the cartilage. The present study was designed to verify the hypothesis described as above by evaluating the influence of Hb on ADAMTS family activity produced by cultured human synovial cells.

## Methods

### Materials

Human Hb: Sigma Chemical Co. (St. Louis, MO, USA); anti-human ADAMTS-5 polyclonal antibody: CEDARLANE Co. (Burlington, Ontario, Canada); Anti-human ADAMTS-8 and -10 polyclonal antibodies: Biorbyt Ltd. (Cambridge, UK); anti-human ADAMTS-9 polyclonal antibody: Sigma; biotinylated link universal streptavidin-HRP secondary antibody and detection system: Dako Co. (Denmark); ELISA kit for ADAMTS-5, −9: Cloud-Clone Corp. (Katy, TX, USA).

### Cell culture

Synovial tissues samples were obtained from five young patients who had confirmed meniscus injuries according to clinical and magnetic resonance imaging findings. All patients had previously undergone arthroscopic surgery due to pain and functional impairment. The mean age of patients was 14 years (range: 10–16).

**Fig. 1:**
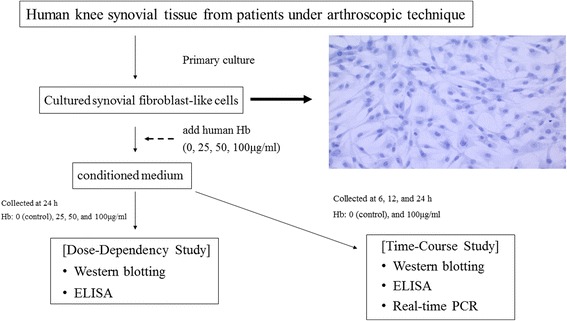
Flowchart of the present study. Human knee synovial tissue was obtained from five patients under arthroscopic surgery. After primary culture, several doses of Hb-treatment were administered. The culture medium was used for Western blotting and ELISA analysis for time-course and dose-dependent studies. Cells were used for real-time PCR analysis of the time-course study. The results shown are typical of several independent experiments (*N* = 6). Hb: hemoglobin. ELISA: Enzyme-Linked Immunosorbent Assay. Real-time PCR: Real-time polymerase chain reaction

Synovial tissues samples were obtained in the operating room under sterile technique and stored in phosphate buffered saline (pH 7.4), immediately. The samples were cut into small pieces at the laboratory under sterile condition, and cultured in T75 flasks (5% CO_2_ in air, 37 °C, pH 7.4), within 1 h of dissection [4]. The culture medium consisted of Dulbecco’s modified Eagle’s medium, nutrient mixture F-12 HAM, 10% heat inactivated fetal calf serum (FCS), penicillin (100 U/ml), and streptomycin (100 mg/ml), according to the previous report [4]. Primary cultures of the samples; human knee synovial tissues were treated with different doses of human Hb (0, 25, 50, 100 μg/ml). The culture media were collected 24 h after Hb-treatment. In the time-course studies, cells were treated with 100 μg/ml Hb and culture media were taken at 6, 12, and 24 h (Fig. 1). The passage 4 or 5 cultured cells were employed in the present study for both Hb-free control and Hb-treated group. Before all investigations, cells were grown in 6-well plates in 2 ml of culture medium without FCS for 24 h. The cultured synovial cells were incubated with various doses of reagents diluted with 1 ml of FCS-free culture medium for the proposed periods. After collection of the conditioned medium, samples were centrifuged at 8000×*g* to remove cell debris, and were stored at −70 °C until assay.

### Western blot analysis

To identify suitable doses of Hb responsible for aggrecanase activity at 24 h, we performed Western blot analysis as a preliminary examination, and to identify suitable incubation times for Hb to cause aggrecanase expression at 100 μg/ml, Western blotting of samples from various time-points was also performed. Samples (10 μl) were applied on SDS-PAGE (10% separating gel, and 5% stacking gel). Broad-range pre-stained protein markers were also applied, and electrophoresis performed for nearly 1.5–2 h in electrode buffer containing of Tris aminomethane, glycine, and 0.1% SDS. After electrophoresis was completed, gels were transferred onto polyvinylidene difluoride membranes, based on the previous report of Burnette [14]. Block Ace was employed for blocking the non-specific binding of immunoglobulin. The transferred membranes were incubated overnight with various primary rabbit anti-human ADAMTS polyclonal antibodies diluted 1:100–500 at 4 °C; ADAMTS-5, −8, −9, and −10, respectively. After washing the membranes extensively, the membranes were incubated with anti-rabbit, mouse, or goat IgG biotinylated link universal streptavidin-HRP secondary antibody for 1 h at room temperature. The Western blotting detection system was employed for detection of protein bands, based on the instructions of manufacture.

### Enzyme-linked Immunosorbent assay (ELISA)

The levels of ADAMTS in the conditioned media were measured by ELISA according to the manufacturer’s instructions.

### Quantitative polymerase chain reaction (real-time PCR)

Total RNA was purified from cultured cells using an RNeasy kit (Qiagen, Valencia, CA, USA), and then converted to cDNA with Moloney murine leukemia virus reverse transcriptase (Invitrogen, Carlsbad, CA, USA). Real-time PCR (Stepone Plus, Applied Biosystems Co., Carlsbad, CA, USA) was performed on each sample for the expression of the ADAMTS-5 and ADAMTS-9 gene. The oligonucleotides used for the PCR amplification of each cDNA are summarized in Table 1 [15, 16]. Endogenous β-actin gene levels were used for normalization and the expression level of each gene was determined using the ΔΔCT method. For relative quantification, results were presented as the relative expression with respect to the value to the 6 h Hb-free control.

**Table 1:** List of primers used for real-time PCR

Target	Sequence (5′ → 3′)
ADAMTS-5 sense	CAAGCGTTTAATGTCTTCAATCCTTA
ADAMTS-5 antisense	ACTGCTGGGTGGCATCGT
ADAMTS-9 sense	GGACAAGCGAAGGACATCC
ADAMTS-9 antisense	ATCCATCCATAATGGCTTCC

### Statistical analysis

Statistical comparisons between each group were performed using paired *t*-tests. Analysis was performed using the statistical software package Ystat 2004 (Igaku Tosho Shuppan Co., Ltd., Tokyo, Japan). The level of significance was set at *P* < 0.05.

## Results

### Western blotting analysis of control and Hb-treated media

**Fig. 2:**
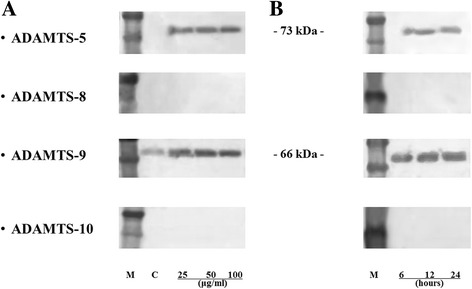
Western blot analysis for ADAMTS-5, −8, −9, and −10 produced by cultured human synovial cells. **a**: Dose-dependency study. Confluent cells were incubated with or without Hb for 24 h, then the conditioned media was collected. M: marker, C: control (without Hb); 25, 50, and 100 μg/ml of Hb were administered. The collected media was analyzed by Western blotting using antibodies against ADAMTS-5, −8, −9, and −10. **b**: Time-course study. Confluent cells were incubated with 100 μg/ml Hb, the conditioned media were collected at 6, 12, and 24 h

To identify the proteins responsible for the aggrecanase expression that appeared 24 h after treatment with varying doses of Hb (25, 50, and 100 μg/ml), we performed Western blotting employing antibodies against human ADAMTS-5, −8, −9, and −10 (Fig. 2a). The results showed that the expression of ADAMTS-5 in Hb-treated media was found as a single band of 73 kDa, and ADAMTS-9 was found as a single band of 66 kDa in both control and Hb-treated medium, although defined dose-dependency effect was not confirmed.

In the time-course study, the immunoreactivity against ADAMTS-5 and -9 in Hb-treated medium was observed as a single band of 73 and 66 kDa, respectively. Peak immunoreactivity was found at 24 h for both ADAMTS-5 and -9 (Fig. 2b). Neither control nor Hb-treated medium showed immunoreactivity against ADAMTS-8 or −10.

### ELISA analysis of control and Hb-treated media: Dose-dependency study

**Fig. 3:**
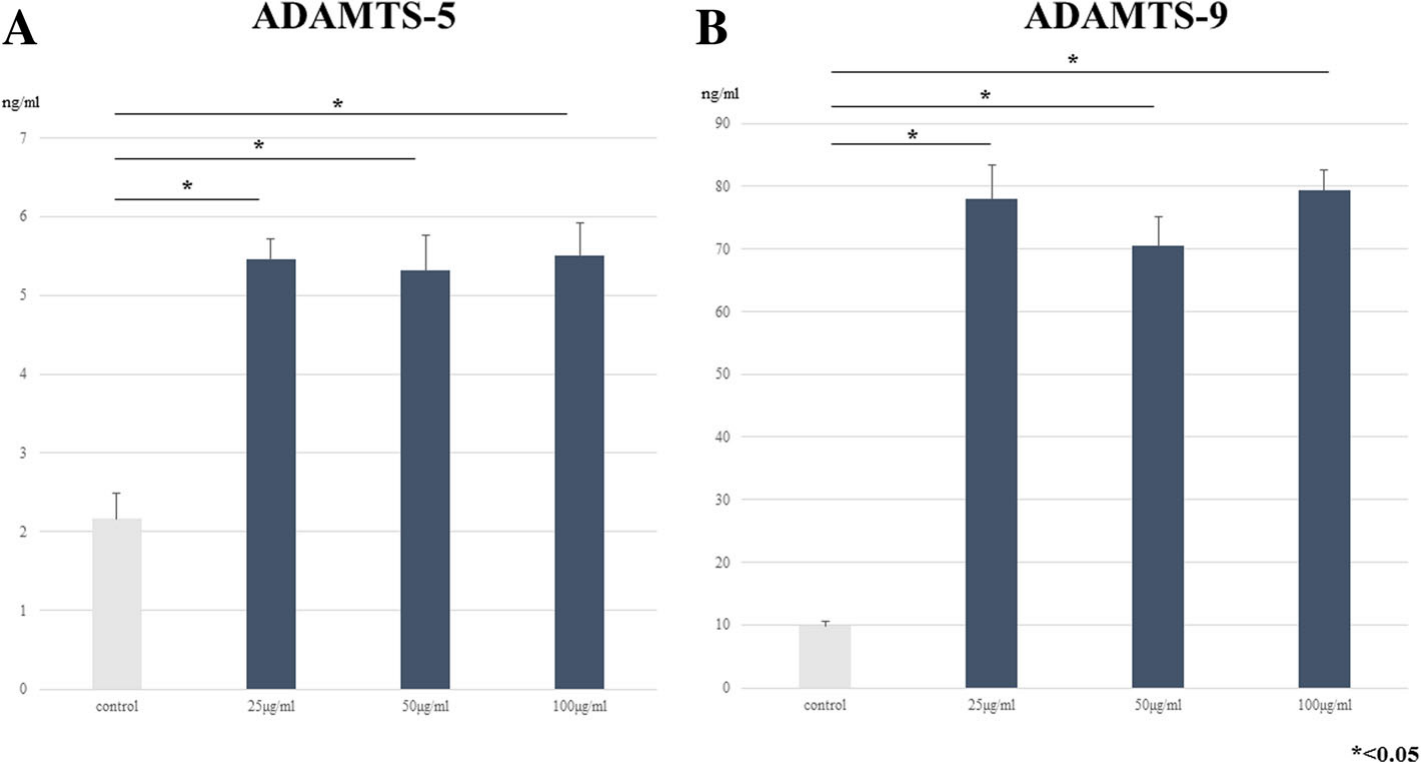
ELISA analysis for dose-dependency study of ADAMTS-5 and -9 produced by cultured human synovial cells. **a**: ELISA analysis for the dose-dependency response of ADAMTS-5 to Hb. Confluent cells were incubated with various concentrations of Hb (0, 25, 50, and100 μg/ml), then the conditioned media were collected at 24 h. The level of ADAMTS-5 significantly increased even at 25 μg/ml compared with the control (*p* < 0.05). **b**: ELISA analysis for the dose-dependent response of ADAMTS-9 to Hb. ADAMTS-9 was highly expressed at 25, 50, and 100 μg/ml significantly after Hb stimulation (*p* < 0.05). There were no Hb dose-dependent effect for ADAMTS-5 and -9 expression by cultured human synovial cells

To identify a suitable dose of Hb to induce aggrecanase activity at 24 h, we performed ELISA analysis for ADAMTS-5 and -9 expression. Levels of ADAMTS-5 and -9 in 25, 50, and 100 μg/ml Hb-treated groups were significantly increased compared with the control, respectively (*p* < 0.05) (Fig. 3a and b). There were no obvious differences between the Hb-treated groups in spite of the different concentrations of Hb.

### ELISA analysis of control and Hb-treated media: Time-course study

**Fig. 4:**
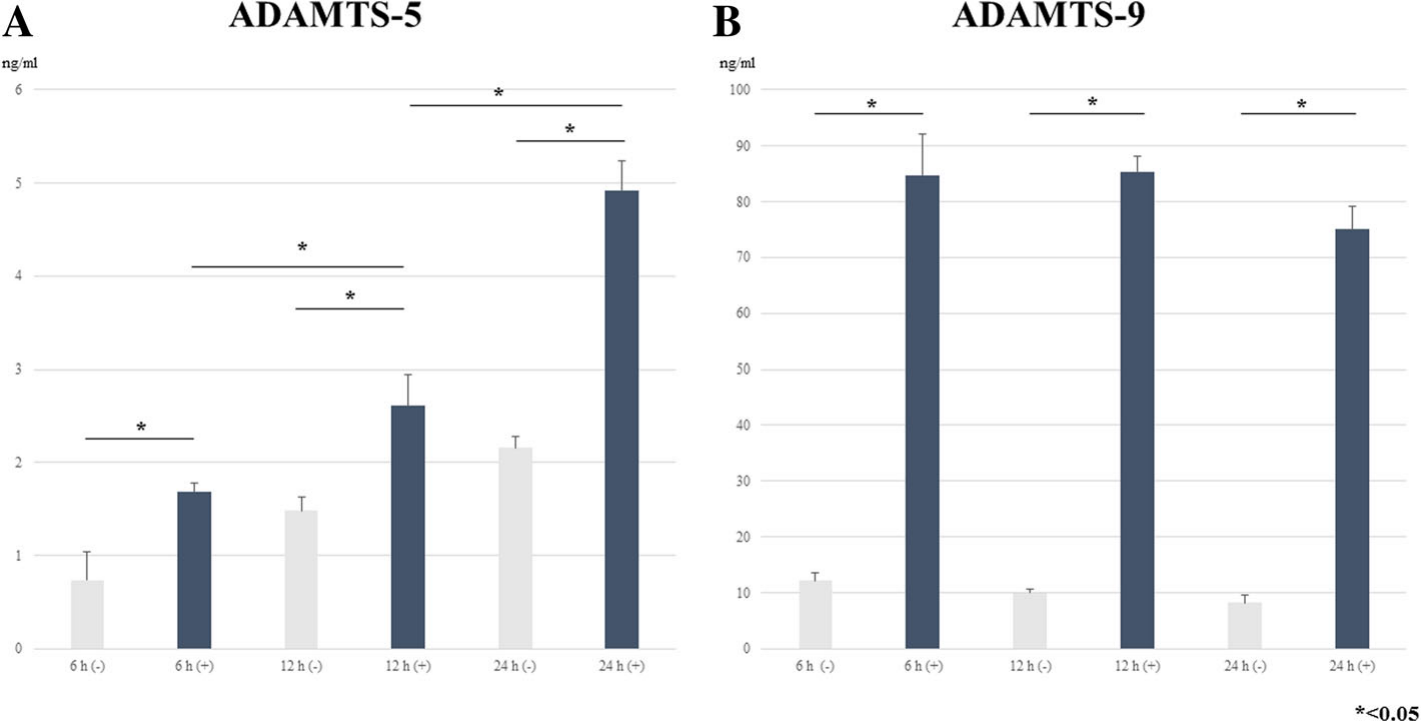
ELISA analysis for time-course study of ADAMTS-5 and -9 produced by cultured human synovial cells. **a**: ELISA analysis for the time-course response of ADAMTS-5 to Hb. Confluent cells were incubated with or without Hb (100 μg/ml), then the conditioned media were collected at 6, 12, and 24 h. The level of ADAMTS-5 showed time-course dependency, and there was significantly higher expression at 6, 12, and 24 h after Hb stimulation (*p* < 0.05). **b**: ELISA analysis for the time-course response of ADAMTS-9 to Hb. ADAMTS-9 was highly expressed at 6, 12, and 24 h significantly after Hb stimulation compared with the control. (*p* < 0.05)

To identify the proteins responsible for aggrecanase activity at 6, 12, and 24 h after Hb-treatment (100 μg/ml), we performed ELISA employing antibodies against human ADAMTS-5 and -9. The levels of ADAMTS-5 and -9 in the conditioned medium revealed significantly increased expression at 6, 12, and 24 h in the Hb-treated group compared with the control group (*p* < 0.05) (Fig. 4a and b). ADAMTS-5 showed time-course dependency in the Hb-treated groups. On the other hand, there was no obvious difference in the results of ADAMTS-9 between 6, 12, and 24-h Hb-treated groups.

### Hb induces ADAMTS gene expression

**Fig. 5:**
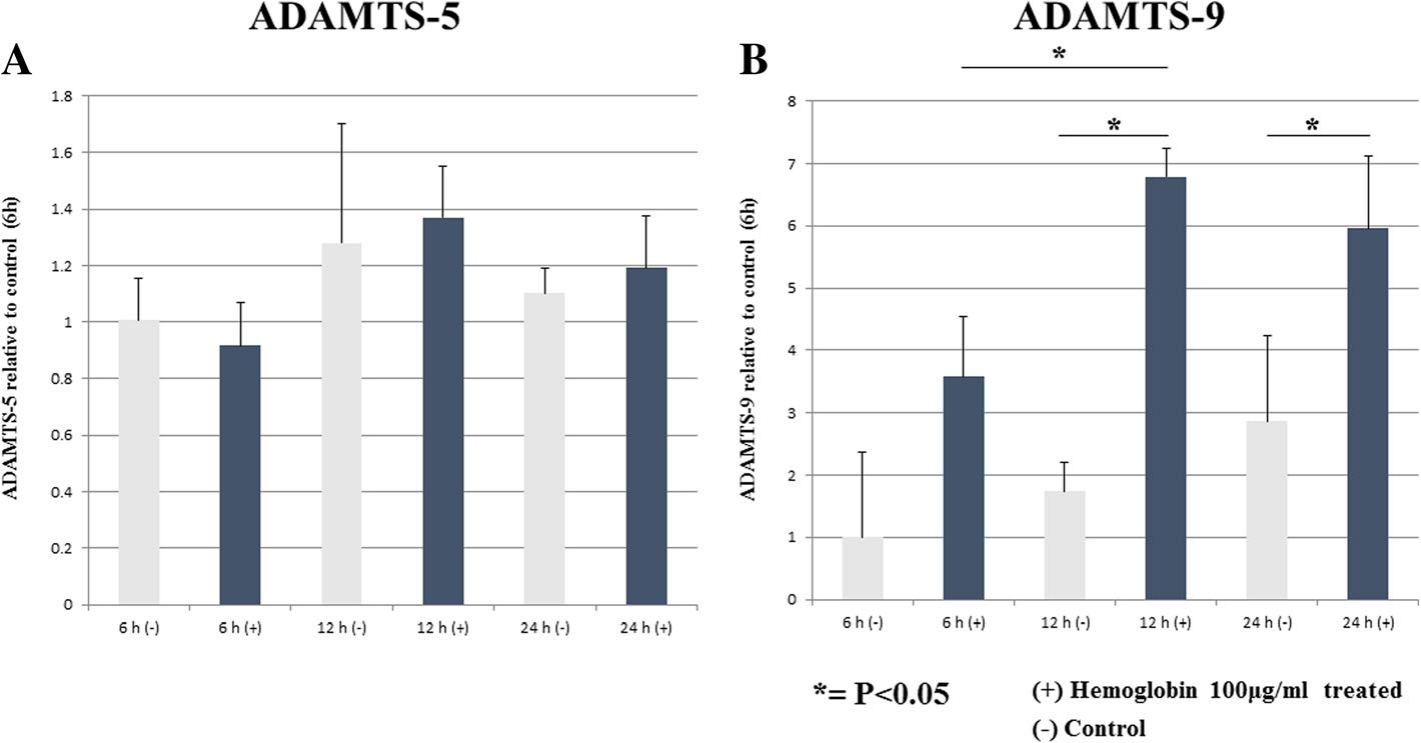
Gene expression of ADAMTS-5 and -9 mRNA. **a**: The time-response of ADAMTS-5 mRNA to Hb. Human synovial cells were cultured in the presence or absence of Hb (100 μg/ml). There were no significant differences between control and Hb treatment groups at each time. **b**: The time-response of ADAMTS-9 mRNA to Hb. ADAMTS-9 mRNA expression was significantly higher at 12 and 24 h after Hb stimulation. (*p* < 0.05)

The mRNA expressions of ADAMTS-5 and -9 upon Hb stimulation were examined in a time-course study. Synovial cells were cultured in the presence and absence of Hb (100 μg/ml), and the results showed that the mRNA levels of ADAMTS-9 were significantly enhanced at 12 and 24 h (*p* < 0.05). The mRNA levels of ADAMTS-5 were not significantly enhanced by Hb (Fig. 5a and b).

## Discussion

Recent studies have found that ADAMTS-5 and -9 play an important pathological role in matrix degeneration; for example, ADAMTS-5 contributes to intervertebral disc and articular cartilage degeneration [10, 11, 17–20], and ADAMTS-9 contributes to arthritis, breast cancer metastasis, and central nervous system pathologies [10, 15, 21, 22]. Moreover, both ADAMTS-5 and -9 cause turnover of proteoglycans in liver fibrosis [23]. Previously, in an animal study, the combined proteolytic activities of ADAMTS-5 and -9 were found to be required keeping extracellular matrix proteolysis above the threshold required for web regression [10]. ADAMTS-8 can cleavage hyaluronan-binding chondroitin sulfate proteoglycan extracellular proteins [10], and that can also exert as an anti-angiogenic factor [10]. Because of such multiple functions and abilities of ADAMTS-8, we sought to investigate the role of ADAMTS-8 in the present study. In contrast, ADAMTS-10 has been reported to contribute to the connective tissue disorder including joint stiffness and cardiac valve stenosis [10]; this protein can bind to fibrillin-1 and -2 and promote microfibril formation in the extracellular matrix, and this function may be independent of protease activity was reported [10]. Therefore, we employed ADAMTS-10 as a contrast of aggrecanase in the present study.

Our preliminary examination found that Hb induced the secretion of ADAMTS-5 and -9 by cultured synovial cells. Dose-dependency studies indicated that Hb induced significant expression of these aggrecanases even at concentrations as low as 25 μg/ml [Fig. 3a and b]. Time-course studies showed that Hb significantly induced the expression of ADAMTS-5 and -9 at 6, 12, and 24 h [Fig. 4a and b]. These enzymes cleave the aggrecanase-specific Glu373-Ala374 bond in the interglobular domain region of aggrecan core protein, and are deeply involved in the pathology of arthritic joint diseases such as osteoarthritis [9–11]. That ADAMTS-9 was observed even in control medium without Hb suggests that it may contribute to normal cartilage turnover. These findings indicate that Hb induce ADAMTS-5 and -9 expressions by human synovial cells at an early phase, even at low doses, after Hb-treatment. We could detect neither ADAMTS-8, which has been reported to cleave aggrecan core protein as well at Glu373-Ala374 [10, 12], nor ADAMTS-10 by Western blotting in the present study [Fig. 2a and b].

Although the pathomechanisms of blood-induced joint degeneration and degradation are still elusive, several pathomechanisms have been suggested via both enzymatic and non-enzymatic processes, such as by expression of neutrophil elastase [24], uPA [4, 25], gelatinases [26], cytokines [27], calpain [28], and hydroxyl radicals [29] produced by inflamed synovial cells and inflammatory cells. Hb is a potent inflammatory substance that can produce a tissue response [30]. It is well known that hydroxyl radicals which can destroy cell membranes were produced by catalytically active iron from Hb via Fenton reaction [31]. On the other hand, there are other pathways that can enzymatically degrade the extracellular matrix of articular cartilage. Of the components of Hb, only the globin chain stimulates fibrinolytic activity, while protoporphyrin IX and heme do not [13]. Once aggrecan was degraded, elasticity of the articular cartilage was disappeared, and the joint shows similar condition to the initial stage of osteoarthritis [8, 9]. The enzymatic depletion of aggrecan is caused by members of the MMPs and ADAMTS family proteins [10, 11, 32]. Previously, ADAMTS-5 was also shown to play an important role in proteoglycan turnover and cartilage degeneration [33, 34]. uPA is able to convert plasminogen to plasmin, which can also cleavage extracellular matrix components [4]. In addition, plasminogen-plasmin system including uPA are also able to activate several MMP family enzymes. Therefore, the combination of the plasminogen-plasmin system, MMPs, and ADAMTS family enzymes could strongly degrade extracellular matrix. Previously, Hb was found to induce the expressions of uPA, MMP-2, and MMP-9 by fibroblasts [4]. Increased levels of aggrecanases, gelatinolytic and fibrinolytic enzymes following Hb stimulation may, at least in part, contribute to degradation of extracellular matrix of articular cartilage.

It has been reported that exposure of human cartilage tissue to low concentrations of blood for as little as 2 days leads to prolonged cartilage damage [3]. Our results suggest that the deleterious effects of Hb on cartilage may occur in an acute phase at 6 h after exposure to blood due to the presence of ADAMTS-5 and -9. The present study aimed to gather information regarding the thresholds of Hb concentration and the time that is responsible for ADAMTS gene expression. We already reported that the peak expressions of gelatinases MMP-2, MMP- 9, and uPA by Hb-treated human synovial cells occurred at 24–48 h [4]. Combined with the results of the present study, it appears that Hb stimulates the expression of MMP-2 and -9, ADAMTS-5 and -9, and uPA from synovial cells in the early stage. The combination of these enzymes may contribute to articular cartilage degeneration after intra-articular hemorrhage.

Our results demonstrated that Hb acutely induced ADAMTS-9 mRNA without enhancing the mRNA level of ADAMTS-5 [Fig. 5a and b]. Although there was a slight time lag when we compared the results of the peak immunoreactivity against ADAMTS-9 with peak mRNA expression, the mRNA expression of ADAMTS-9 at 12 and 24 h after Hb-treatment was significantly higher than that of the control [Fig. 5b]. Previously, Yoshida et al. reported that Hb enhances the synthesis of uPA through post-transcriptional regulation of mRNA [13]. Moreover, ADAMTS-5 is expressed constitutively in synovium with no or little transcriptional regulation when activated by recombinant human interleukin-1 alpha [35]. Thus, it is possible that separate stimulation pathways and mechanisms could exist between ADAMTS-5 and -9 when they are treated by Hb. Further investigations to clarify the mechanism by which Hb stimulates the expression of ADAMTS family members in vivo and in vitro are required.

It is well known that two major proteinases in the ADAMTS family are the capital enzymes contributed in the pathogenesis of arthritis: ADAMTS-4 and ADAMTS-5 called aggrecanase-1 and aggrecanase-2, respectively [8–11, 36]. Recently, synovial fibroblasts have been reported to provide the higher levels of ADAMTS-4 in osteoarthritis [37]. Although these enzymes are the principal aggrecanases those present in cartilage, previous in vitro study showed ADAMTS-5 is 1000-times more effective than ADAMTS-4 [38]. In animal model of arthritis, ADAMTS-5 alone is the critical enzyme to contribute for joint destruction were also reported [39, 40]. Thus, ADAMTS-5 may be an important target for therapeutic inhibition to prevent articular cartilage degeneration.

New therapies based on reducing or inhibiting the levels of ADAMTS-5 using an anti-ADAMTS-5 antibody in animal models of osteoarthritis have been reported [34, 41, 42]. Given the results of the present study, we suggest that provision of both anti-ADAMTS-5 and -9 antibodies into the joint at the early phase after intra-articular hemorrhage could be a superior therapeutic option to prevent the cartilage degeneration.

## Conclusions

Our results suggest a possible role for Hb in joint damage after intra-articular hemorrhage. Hb induces the expression of ADAMTS family proteinases such as ADAMTS-5 and -9 by synovial cells at low doses even at an acute phase. These findings suggest that a single instance of intra-articular hemorrhage during trauma or sports injury could deleteriously affect articular cartilage metabolism. Immediate treatment for intra-articular hemorrhage, such as washing out and removing the blood from the joint cavity, may be required regardless of whether it occurs once or multiple times. Moreover, it could be possible that inhibiting the activities of ADAMTS-5 and -9 together may serve as a superior therapeutic strategy to prevent cartilage damage after intra-articular hemorrhage.

## Abbreviations

ADAMTS: A disintegrin and metalloprotease with thrombospondin motifs
ELISA: Enzyme-Linked Immunosorbent Assay
Hb: Hemoglobin
MMP: Matrix metalloproteinase
mRNA: messenger ribonucleic acid
Real-time PCR: Real-time polymerase chain reaction
uPA: urokinase type plasminogen activator

## References

[1] Madhok R, Bennett D, Sturrock RD, Forbes CD (1988). Mechanisms of joint damage in an experimental model of hemophilic arthritis. Arthritis Rheum.

[2] Rodriguez-Merchan EC (1997). Pathogenesis, early diagnosis, and prophylaxis for chronic hemophilic synovitis. w.

[3] Jansen NW, Roosendaal G, Bijlsma JW, DeGroot J, Lafeber FP (2007). Exposure of human cartilage tissue to low concentrations of blood for a short period of time leads to prolonged cartilage damage. Arthritis Rheum.

[4] Tajima T, Yoshida E, Yamashita A (2005). Hemoglobin stimulates the expression of matrix metalloproteinases, MMP-2 and MMP-9 by synovial cells: a possible cause of joint damage after intra-articular hemorrhage. J Orthop Res.

[5] Roosendaal G, TeKoppele JM, Vianen ME, van den Berg HM, Lafeber FP, Bijlsma JW. Blood-induced joint damage; a canine in vivo study. Arthritis Rheum 1999: 42: 1033–1039. 10.1002/1529-0131(199905)42:5<1033::AID-ANR24>3.0.CO;2-# 10323461

[6] Roosendaal G, Vianen ME, Marx JJ, van den Berg HM,, Lafeber FP, Bijlsma JW. Blood-induced joint damage; a human in vitro study. Arthritis Rheum 1999: 42: 1025–1032. 10.1002/1529-0131(199905)42:5<1025::AID-ANR23>3.0.CO;2-3 10323460

[7] Roosendaal G, Vianen ME, van den Berg HM, Lafeber FP, Bijlsma JW. Cartilage damage as a result of hemarthrosis in a human in vitro model. J Rheumatol 1997: 24: 1350–1354. 9228136

[8] Huang K, Wu LD (2008). Aggrecanase and aggrecan degradation in osteoarthritis: a review. J Int Med Res.

[9] Verma P, Dalal K (2011). ADAMTS-4 and ADAMTS-5: key enzyme in osteoarthritis. J Cell Biochem.

[10] Kelwick R, Desanlis I, Wheeler GN, Edwards DR (2015). The ADAMTS (a Disintegrin and metalloproteinase with Trombospondin motifs) family. Genome Biol.

[11] Lin EA, Liu CJ (2010). The role of ADAMTSs in arthritis. Protein & Cell.

[12] Tortorella MD, Malfait AM (2008). Will the real aggrecanase(s) step up: evaluating the criteria that define aggrecanase activity in osteoarthritis. Curr Pharm Biotechnol.

[13] Yoshida E, Ohmura S, Sugiki M, Anai K, Maruyama M (2001). A novel function of extraerythrocytic hemoglobin: identification of globin as a stimulant of plasminogen activator biosynthesis in human fibroblasts. Thromb Haemost.

[14] Burnette WN (1981). 'Western blotting': electrophoretic transfer of proteins from sodium dodecyl sulfate-polyacrylamide gels to unmodified nitrocellulose and radiographic detection with antibody and radiodinated protein A. Anal Biochem.

[15] Demican K, Hirohata S, Nishida K (2005). ADAMTS-9 is synergistically induced by interleukin-1 beta and tumor necrosis factor alpha in OUMS-27 chondrosarcoma cells and in human chondrocytes. Arthritis Rheum.

[16] Voros G, Sandy JD, Collen D, Lijnen HR (1760). Expression of aggrecan(ases) during murine preadipocyte differentiation and adipose tissue development. Biochim Biophys Acta.

[17] Binch AL, Shapiro IM, Risbud MV (2016). Syndecan-4 in intervertebral disc and cartilage: saint or Synner?. Matrix Biol.

[18] Larkin J, Lohr TA, Elefante L (2015). Translational development of an ADAMTS-5 antibody for osteoarthritis disease modification. Osteoarthr Cartil.

[19] Liu YD, Yang HX, Liao LF (2016). Systemic administration of strontium or NBD peptide ameliorates early stage cartilage degradation of mouse mandibular condyles. Osteaarthritis Cartilage.

[20] Ye F, Wang H, Zheng Z, et al. Role of SHOX2 in the development of intervertebral disc degeneration. J Orthop Res. 2015 10.1002/jor.23140. 10.1002/jor.23140 26697824

[21] Ocak Z, Acar M, Gunduz M, Demican K, Uyeturk U, Ozlu T (2013). Effect of hypericin on the ADAMTS-9 and ADAMTS-8 gene expression in MCF7 breast cancer cells. Eur Rev Med Pharmacol Sci.

[22] Reid MJ, Cross AK, Haddock G, et al. ADAMTS-9 expression is up-regulated following transient middle cerebral artery occlusion (tMCAo) in the rat. Neurosci Lett 2009: 20. 452:252–7. 10.1016/j.neulet.2009.01.058 19348733

[23] Bukong TN, Maurice SB, Chahal B, Schaeffer DF, Winwood PJ (2016). Versican: a novel modulator of hepatic fibrosis. Lab Investig.

[24] Hilbert N, Schiller J, Arnhold J, Arnold K (2002). Cartilage degradation by stimulated human neutrophils: elastase is mainly responsible for cartilage damage. Bioorg Chem.

[25] He B, Shi C-H, Wang Y-M, Jiang XZ, Sun JH, Shi H (2007). Effects of urokinase-type plasminogen activator on articular cartilage damage. J Clin Rehabilitative Tissue Eng Res.

[26] Van den Steen PE, Proost P, Grillet B (2002). Cleavage of denatured collagen type 2 by neutrophil gelatinase B reveals emzyme specificity, post-translational modifications in the substrate, and the formation of remnant epitope in rheumatoid arthritis. FASEB J.

[27] Malcolm DS, Triantafillou S, Parker A, Youssef PP, Coleman M (1997). Synovial membrane inflammation and cytokine production in patients with early osteoarthritis. J Rheumatol.

[28] Yamamoto S, Shimizu K, Shimizu K, Suzuki K, Nakagawa Y, Yamamuro T (1992). Calcium-dependent cysteine proteinase (Calpain) in human arthritic synovial joint. Arthritis Rheum.

[29] Burkhardt H, Schwingel M, Menninger H, Macartney HW, Tschesche H (1986). Oxygen radicals as effectors of cartilage destruction. Arthrisis Rheum.

[30] Zardeneta G, Milam SB, Schmitz JP (2000). Iron-dependent generation of free radicals: plausible mechanisms in the progressive deterioration of the temporomandibular joint. J Oral Maxillofac Surg.

[31] Sadrzadeh SMH, Graf E, Panter SS, Hallaway PE, Eaton JW. Hemoglobin: a biologic Fenton reagent. J Biol Chem. 1984;1984(259):14354–6. 6094553

[32] Struglics A, Larsson S, Pratta MA, Kumar S, Lark MW, Lohmander LS (2006). Human osteoarthritis synovial fluid and joint cartilage contain both aggrecanase- and matrix metalloproteinase-generated aggrecan fragments. Osteoarthr Cartil.

[33] Didangelos A, Mayr U, Monaco C, Mayr M (2012). Novel role of ADAMTS-5 protein in proteoglycan turnover and lipoprotein retention in atherosclerosis. J Biol Chem.

[34] Miller RE, Tran PB, Ishihara S, Larkin J, Malfait AM (2016). Therapeutic effects of an anti-ADAMTS-5 antibody on joint damage and mechanical allodynia in a murine model of osteoarthritis. Osteoarthr Cartil.

[35] Vankemmelbeke MN, Holen I, Wilson AG (2001). Expression and activity of ADAMTS-5 in synovium. Eur J Biochem.

[36] Song RH, Tortorella MD, Malfait AM (2007). Aggrecan degradation in human articular cartilage explants is mediated by both ADAMTS-4 and ADAMTS-5. Arthritis Rheum.

[37] Pérez-García S, Gutiérrez-Cañas I, Seoane IV (2016). Healthy and osteoarthritic synovial fibroblasts produce a disintegrin and metalloproteinase with thrombospondin motifs 4, 5, 7, and 12: induction by IL-1β and fibronectin and contribution to cartilage damage. Am J Pathol.

[38] Gendron C, Kashiwagi M, Lim NH (2007). Proteolytic activities of human ADAMTS-5: comparative studies with ADAMTS-4. J Biol Chem.

[39] Glasson SS, Askew R, Sheppard B (2005). Deletion of active ADAMTS5 prevents cartilage degradation in a murine model of osteoarthritis. Nature.

[40] Stanton H, Rogerson FM, East CJ (2005). ADAMTS-5 is the major aggrecanase in mouse cartilage *in vivo* and *in vitro*. Nature.

[41] Apte SS (2016). Anti-ADAMTS5 monoclonal antibodies: implications for aggrecanase inhibition in osteoarthritis. Biochem J.

[42] Santamaria S, Yamamoto K, Botkjaer K (2015). Antibody-based exosite inhibitors of ADAMTS-5 (aggrecanase-2). Biochem J.

